# Estimating utilities/disutilities for high-risk metastatic hormone-sensitive prostate cancer (mHSPC) and treatment-related adverse events

**DOI:** 10.1007/s11136-019-02117-9

**Published:** 2019-02-14

**Authors:** F. Hall, H. M. de Freitas, C. Kerr, T. Ito, B. Nafees, A. J. Lloyd, J. Penton, M. Hadi, S. Lanar, T. P. Pham

**Affiliations:** 1 Janssen UK, High Wycombe, UK; 2 Patient Centered Sciences, Mapi, London, UK; 3 Janssen EMEA, High Wycombe, UK; 4 Nafees Consulting Ltd, London, UK; 5 Acaster Lloyd Consulting Ltd, London, UK; 6 Patient Centered Sciences, Mapi, Lyon, France; 7 Patient Centered Sciences, Mapi, Boston, USA

**Keywords:** Utility, Disutility, Metastatic hormone-sensitive prostate cancer, Androgen deprivation therapy, Docetaxel

## Abstract

**Purpose:**

To capture UK societal health utility values for high-risk metastatic hormone-sensitive prostate cancer (mHSPC) and the disutility associated with treatment-related adverse events (AEs) to inform future cost–utility analyses.

**Methods:**

A literature review, and patient and clinical expert interviews informed the development of health states characterising mHSPC symptoms and the impact of treatment-related AEs on health-related quality of life (HRQL). Three base health states were developed describing a typical patient with high-risk mHSPC: receiving androgen deprivation therapy (ADT) [Base State 1]; receiving docetaxel plus ADT [Base State 2]; completed docetaxel and still receiving ADT whose disease has not yet progressed [Base State 3]. Six additional health states described treatment-related AEs. The health states were validated with experts and piloted with general public participants. Health state utilities were obtained using the time trade-off (TTO) method with 200 members of the UK general population. A generalised estimating equation (GEE) model was used to estimate disutility weights.

**Results:**

Mean TTO scores for Base State 1 to 3 were 0.71 (SD = 0.26), 0.64 (SD = 0.27), and 0.68 (SD = 0.26), respectively, indicating that receiving docetaxel plus ADT was most impactful on HRQL. The GEE model indicated when compared to Base State 2 that the nausea and vomiting AE had the most impact on HRQL (− 0.21), while alopecia was least burdensome (− 0.04).

**Conclusions:**

The study highlights the differences in utility between base health states and the significant impact of treatment-related AEs on the HRQL of patients with mHSPC. These findings underline the importance of accounting for impaired HRQL when assessing treatments for mHSPC.

**Electronic supplementary material:**

The online version of this article (10.1007/s11136-019-02117-9) contains supplementary material, which is available to authorised users.

## Introduction

Prostate cancer is the most common male cancer in the UK with over 47,000 men diagnosed every year [1]. Although it typically affects older men, aged between 65 and 79 years, approximately a quarter of all cases affect men under the age of 65 [2]. Men who present with metastases at initial diagnosis of prostate cancer are classified as having newly diagnosed metastatic hormone-sensitive prostate cancer (mHSPC), which currently accounts for approximately 18% of cases in the UK [1]. Disease severity at diagnosis is assessed according to several prognostic factors that are associated with poorer survival. Patients could be considered as having ‘high-risk’ disease if they have two of the following three risk factors: a Gleason score of ≥ 8 (describing the aggressiveness of disease), the presence of three or more lesions on a bone scan, or the presence of visceral metastases (describing the extent of disease) [3].

Symptoms of metastatic prostate cancer commonly include bone pain, fatigue, and urinary complications, as well as the psychological effects associated with both the impact of diagnosis and, later, disease progression [4]. There are also numerous side effects associated with current treatment, such as sexual dysfunction, fatigue, and gastrointestinal issues [4–7]. The burden of disease and the potential impact of treatment mean that men with mHSPC often experience an impaired health-related quality of life (HRQL) [4].

Health technology assessments (HTAs) are commonly undertaken to provide recommendations regarding the appropriate use of novel treatments. To support the decision-making process, many HTA authorities utilise cost-effectiveness analysis for measuring health benefits and costs. Change in a patient’s health state and the benefits/risks of treatments should be expressed in terms of quality-adjusted life years (QALYs). QALYs reflect changes in both the quantity and quality of life associated with an intervention or health technology. A measure of health status (health utilities) are conventionally anchored at 0 (dead) and 1 (full health), with potential values < 0 for health states considered to be worse than being dead. Health status should ideally be reported directly from patients, and the derived utilities should be based on societal preferences using a choice-based method such as time trade-off (TTO) [8]. Given the need for consistency across appraisals, the National Institute for Health and Care Excellence (NICE) recommends utilities derived from patients completing the EQ-5D measure (which has societal weights) [9]. However, even when the EQ-5D has been included in the relevant clinical trials, it is not uncommon for trials to fail to capture all the data required for a cost-effectiveness model [10]. Adverse events (AEs) for oncology treatments can be very difficult to assess in terms of their impact on HRQL for a number of practical reasons, but they are important to include in decision models. An alternative approach to EQ-5D for deriving utilities is the ‘vignette’ method, in which utility values are elicited from members of the general public using a choice-based method, e.g. TTO, to value descriptions of health states (known as vignettes). The ‘vignette’ approach is a departure from the NICE reference case (i.e. patients who completed the EQ-5D) but can be useful for eliciting utilities in situations in which obtaining valid and direct reports from a sufficient number of patients can be challenging. This can occur in cases of relatively rare conditions, in disease sub-groups or when evaluating the impact of treatment AEs on HRQL.

Androgen deprivation therapy (ADT) has been the standard of care for men with mHSPC for the past few decades, either by means of surgical castration (orchiectomy) or medical castration [11]. Although most patients initially respond to treatment, the majority develop progressive disease within 1–2 years of diagnosis which is resistant to further ADT and therefore defined as metastatic castration-resistant prostate cancer (mCRPC) [12]. Docetaxel is now used in combination with ADT as the standard of care to improve survival in men who are eligible for chemotherapy, particularly those with a high metastatic burden [3, 13, 14]. There is, however, a lack of data describing the impact on HRQL in patients with mHSPC and currently no published health utility data describing the impact on patients in this setting receiving docetaxel plus ADT. There are published utility values for prostate cancer in the metastatic castrate-resistant setting [15]; however, as this is distinctly different from the mHSPC setting, it is important to understand the impact on HRQL specifically in patients with mHSPC. The aim of this study was to capture societal-based utility values for health states related to mHSPC and disutility values for AEs associated with active treatment which could be used as inputs to cost–utility analyses.

## Methods

### Literature review

To understand the impact of mHSPC and its available treatments on HRQL, a targeted literature review was conducted with a focus on identifying studies that used qualitative methods. Given the health states of interest to this study, this literature review specifically focused on mHSPC treated with ADT with or without docetaxel. The literature search was performed on the MedLine, Embase, and PsycInfo databases in November 2016 and identified 44 abstracts (See Supplementary Material for detailed search strategies). Additional articles were identified following a free text search of PubMed and Google Scholar. The searches resulted in the identification of three articles which examined patients with mHSPC treated with ADT [4, 6, 7], and five studies which evaluated patients with mHSPC treated with docetaxel [4, 13, 14, 16, 17]. Patients with metastatic prostate cancer reported pain as a frequent symptom which impacted their daily functioning, as well as fatigue, weight loss, depression, and urinary symptoms [4]. The most common side effects of ADT were reported as fatigue, which impacted social activities and exercise, hot flushes, breast growth, psychological burden [4, 6], and sexual dysfunction [7]. Patients receiving docetaxel reported experiencing fatigue, hair loss, bone pain, loss of appetite, weight loss, nausea, and detrimental emotional impact [4].

### Information interviews

Information interviews were conducted by telephone with patients with mHSPC (*n* = 4), clinicians (*n* = 3), and specialist nurses (*n* = 2). Patients were recruited via a patient association, Tackle Prostate Cancer. Written informed consent was obtained before conducting the interviews. Eligible patients had had a recent diagnosis of mHSPC (ideally within the last 7 months), were currently receiving ADT alone or docetaxel plus ADT, aged 18 or over, a resident in the UK, able to speak English fluently, and able and willing to provide informed consent.

Health care professionals (HCPs) were recruited through a specialist recruitment agency and had to be a qualified urologist, oncologist, or specialist nurse with at least 2 years of experience treating patients with mHSPC. The patients were asked about their symptoms, their experience of ADT and docetaxel, and the impact of the disease on their HRQL. HCPs were asked similar questions related to their experience of treating mHSPC patients.

The mHSPC patients in this sample reported experiencing few symptoms other than urinary symptoms; conversely, the HCPs described fatigue and bone pain as being very common symptoms in this population, with the latter impacting daily activities, mobility, and sleep. However, the patients interviewed were not necessarily high-risk, while the HCPs were asked to consider their experience of high-risk patients when responding to questions. The AEs experienced by patients in relation to ADT were reported as hot flushes and sexual dysfunction. The HCPs also described fatigue and depression in patients taking ADT, with breast tenderness and sexual dysfunction as the most burdensome AEs. Of the two patients who had experienced docetaxel, one reported experiencing diarrhoea, alopecia, and neutropenia, and the other avoided going out during the week of infusion to reduce the risk of infection. The HCPs said that fatigue, the risk of infection, and nausea were the most impactful AEs to patients.

### Health state development

Three base health states were defined which represented clinically relevant stages of the patient pathway:

- Base Health State 1: A typical patient diagnosed with high-risk metastatic prostate cancer who is currently receiving ADT and is not yet castrate-resistant.
- Base Health State 2: A typical patient with high-risk metastatic prostate cancer who is currently receiving docetaxel plus ADT and is not yet castrate-resistant.
- Base Health State 3: A typical patient with high-risk metastatic prostate cancer who has completed six cycles of docetaxel, who is currently on ADT, is not yet castrate-resistant and has not yet progressed.

Draft descriptions of the health states were informed by the literature and information interviews, using language which was considered appropriate for members of the general public with no specific medical knowledge. The health state descriptions were structured to describe the disease and its treatment, followed by symptoms and the impact of the disease and treatment across HRQL, using the dimensions measured in the EQ-5D (mobility, self-care, usual activity, pain/discomfort, anxiety/depression).

In order to obtain values for the disutility of the AEs associated with treatment, descriptions of six AE states were developed. Relevant AEs and their respective grading were identified for valuation by reviewing the safety data from the results of relevant clinical trials [13, 14], the Summary of Product Characteristics for treatments [18, 19], and the feedback from the information interviews. The Common Terminology Criteria for Adverse Events (CTCAE), version 4.0 [20] was used to describe grading and definitions:

- Fatigue: (*Grade 3*): Fatigue not relieved by rest; limiting self-care activity of daily living (ADL)
- Nausea and vomiting: (*Grade 3*–*4*): Nausea: Inadequate oral caloric or fluid intake; IV fluids, tube feedings, or TPN indicated ≥ 24 h/vomiting: ≥ 6 episodes in 24 h; IV fluids, or TPN indicated ≥ 24 h
- Reduced immunity and higher susceptibility to infections (*Grade 3*–*4*): neutrophils < 1000/mm^3^ to 500/mm^3^ or neutrophils < 500/mm^3^
- Fluid retention (*Grade 3*): >30% inter-limb discrepancy in volume; gross deviation from normal anatomic contour, limiting self-care ADL
- Alopecia (*Grade 2*): Hair loss of ≥ 50 percent normal for that individual that is readily apparent to others; a wig or hair piece is necessary if the patient desires to completely camouflage the hair loss; associated with psychosocial impact
- Diarrhoea (*Grade 3*–*4*): Increase of seven or more stools per day over baseline; incontinence; hospitalisation indicated; severe increase in ostomy output compared with baseline; limiting self-care activities of daily living/life-threatening consequences; urgent intervention indicated.

All AE descriptions were separately integrated within the wording of Base State 2 to avoid bias in the valuation and to ensure they were valued consistently by the respondents. As Base State 2 was expected to represent the lowest utility of the three base health states, the magnitude of the derived disutility was anticipated to be conservative. Even though some AEs may be more commonly associated with one treatment than with another, all AEs could be experienced in relation to docetaxel. Therefore, valuing them in relation to Base State 2 had face validity.

### Health state validation and revision

The nine health state descriptions (three base states and six AE states each integrated with Base State 2) were validated through interviews with clinicians (*n* = 5) and a specialist nurse (*n* = 1) who had not been involved with the study previously. The HCPs were asked to evaluate the health states to ensure that they accurately reflected the experience of a typical patient with high-risk mHSPC and the impact of treatment on the AEs experienced at the specified CTCAE severity grading. Revisions to wording of symptoms or AEs were made as a result of the HCPs’ feedback.

The health states were also piloted with members of the UK general public (*n* = 5) in face-to-face interviews to assess their understanding of the vignettes. Pilot feedback indicated the health states were well understood and only one minor change was implemented.

### Health state valuation

A sample of the UK general public (aged 18+ and currently resident in the UK) was invited to participate in the valuation study. Eligible participants were recruited using methods such as local advertising and word-of-mouth (snowball sampling) across geographical areas in the UK, including London, Bristol, Edinburgh, and Argyll. The participants were enrolled based on their socio-demographic characteristics to approximate the UK general public (a target percentage of the sample was set with respect to characteristics such as gender and age). The recruitment agency stopped recruitment once the quota of 200 respondents was reached.

Participants in the study provided written informed consent, demographic data, and health status (EQ-5D-5L) data [21, 22]. Face-to-face interviews were conducted in a meeting room by trained interviewers using a standardised interview script, cards with printed health state descriptions, and other props for the VAS (feeling thermometer) and TTO exercises (TTO board).

The participants first undertook a ranking exercise in which the 9 health states and a dead state were rated from most-to-least-preferred using a 100-point visual analogue scale (VAS). The scale ranged from 0 (“worst health”) to 100 (“best health”). This task provided an indication of whether any states were considered worse than dead. All health states were identified using only a simple two-letter code; no reference was made to the name of the health state. The interviewer shuffled the cards so that the order health states were presented varied from one participant to another.

The TTO exercise began by rating health states that were assessed as “better than dead” (i.e. scored above “dead” on the VAS rating exercise) [23]. Participants were asked to imagine that they were in the selected health state then chose between (1) to live in the health state without improvement for 10 years, followed by death or (2) to live in 10 − *x* years of full health followed by death, or (3) to indicate that the two life options were equally desirable. The process incorporated a ‘ping-pong’ approach with the time in full health traded back and forth between higher and lower values that were iteratively narrowed until the participant indicated that they were indifferent between the two life choices (option 3).

For states rated as ‘worse than dead’ on the VAS rating exercise or if the participant preferred death (0 years in full health) to 10 years in the health state, the participant was first asked to confirm that they considered the state as worse than dead. The interviewer then switched to the lead-time approach for worse than dead states only [24]. In the lead-time TTO (LT-TTO), the participant is asked to choose between (1) to live 10 years of full health (lead-time) then 10 years in the health state followed by death, or (2) to live 20 − *x* years in full health (10 years lead-time followed by up to another 10 years of full health), followed by death, or (3) to indicate that the two options were equally desirable (indifferent between the two life choices). The exercise follows the same ‘ping-pong’ approach as the standard TTO and at the point of indifference, whether or not the number of years of full health left in option 2 is greater or less than 10 years (lead-time) indicates a state valued as better or worse than dead. The utility scores were obtained using a score sheet according to the TTO measurement and valuation of health (MVH) protocol [25].

### Statistical analysis

Socio-demographics, EQ-5D-5L data, as well as TTO and VAS scores were summarised using descriptive analysis and compared with the characteristics of the 2011 UK census [26] to assess representativeness of the UK population.

Generalized estimating equation (GEE) models were used to determine the difference in utility score between stages of treatment (ADT alone [Base State 1], docetaxel plus ADT [Base State 2], and post-docetaxel [Base State 3]), as well as understanding the disutility due to AEs. GEE takes into account that there are multiple health state valuations reported by the same participants, unlike for traditional regression methods, the assumption of independence is not required for GEE models. A random effect at the participant level was included in this model to account for the correlation between different TTO valuations by the same participant. The distribution of all TTO values was left-skewed. Potential transformations, such as square-root, squared, power of three and centring around the median, were tested. The raw TTO values were transformed by raising the TTO values by a power of three (transformed utility = utility^3^) as this transformed distribution showed a better fit for the GEE model than the non-transformed distribution and so reduced skewness.

For the GEE models, Base State 1 was the reference state for determining the difference of utility across base states, while Base State 2 was the reference state for understanding AE disutilities. The transformed utility values were used as the response variable and AEs or base states as a predictor variable. An additional GEE model was run to test and adjust for potential explanatory sample variables: participant’s age, gender, and EQ-5D index score. The goodness of fit for both AE GEE models (unadjusted and adjusted) was examined by using the quasilikelihood under the independence model criterion (QIC) statistics. All data processing and analyses were performed with SAS^®^ software for Windows Version 9.4 (SAS Institute, Inc., Cary, NC, USA).

## Results

### Study participants

Socio-demographic characteristics of the 200 study participants are presented in Table 1. The sample was diverse in terms of gender, age, ethnicity, level of education, economic and marital status. Based on population statistics for England and Wales [26], the sample was broadly representative, although with a 5% lower proportion of men (44% vs. 49%), 5-year lower median age (35 years vs. 40 years), a higher proportion with degree-level or higher educational qualification (54% vs. 27%), and higher proportions of non-white ethnic groups, although still mainly of white ethnicity (71% vs. 86%). On the EQ-5D-5L items, the majority of participants reported no problems with mobility (87%), self-care (95%), or performing their usual activities (89%), no pain or discomfort (70.5%) and were not anxious or depressed (71.5%). Participants had a high mean VAS (83.43) and a high mean index score (0.93) indicating good overall health on average. This compares to mean index scores previously published for the UK population of 0.86, with higher population values from people aged below 45 years (in the 0.91–0.94 range) [27].

**Table 1:** Participants’ characteristics (*N* = 200)

Variable	All participants (*n* = 200) % (*n*)
Gender, *n*	
Male/female	44.0/56.0 (88/112)
Age, years	
Mean (SD)	35.2 (12.3)
Median	33.0
Min–max	18.0–79.0
Highest level of education, *n*	
Left school with no qualifications	2.5 (5)
Left school with qualifications	22.5 (45)
Completed some college	19.5 (39)
Degree/postgraduate level	54.0 (108)
Missing	1.5 (3)
Main activity, *n*	
Employed full-time	49.5 (99)
Employed part-time	14.0 (28)
Seeking work	2.0 (4)
Student	17.0 (34)
Retired	4.5 (9)
Unemployed	4.0 (8)
Other	8.0 (16)
Missing	1.0 (2)
Marital status	
Single	44.5 (89)
Partnership	25.0 (50)
Married	26.0 (52)
Divorced/separated	4.5 (9)
Ethnicity	
White	70.5 (141)
Mixed race	7.0 (14)
Asian	10.5 (21)
Black, African or Caribbean	6.5 (13)
Chinese or other ethnic groups	1.5 (3)
Prefer not to answer	3.5 (7)
Missing	0.5 (1)
EQ-5D VAS	
Mean (SD)	83.4 (13.0)
Median	85.0
Min–max	20.0–100.0
EQ-5D index score	
Mean (SD)	0.93 (0.13)
Median	1.0
Min–max	− 0.11 to 1.0

*Max* maximum, *Min* minimum, *n* number, *SD* standard deviation, *VAS* visual analogue scale

### Descriptive summary of results

Values from the VAS and TTO exercises for all health states (base and AE health states) are presented in Table 2. Utility values that were derived from the TTO exercise were aligned with the rating of the health states on the VAS. Health states with higher utility values were rated higher on the VAS. All states were rated quite severely: the mean VAS scores for all states were below 50. Base State 1 had the highest mean TTO score (0.71 ± 0.26), and Base State 2 resulted in the lowest TTO score (0.64 ± 0.27), indicating that this was considered the worst of the base health states. Overall, the utility of the AE state of fluid retention was ranked the highest (mean TTO value of 0.58 ± 0.29), while diarrhoea was ranked the lowest (mean TTO value of 0.40 ± 0.38). Diarrhoea was therefore considered most impactful on the quality of life to responders.

**Table 2:** Observed values from VAS and TTO exercises for all health states

Health state	VAS value (*n* = 200) Mean (SD)	TTO value (*n* = 200) Mean (SD)
Base State 1 (receiving ADT)	49.4 (19.1)	0.71 (0.26)
Base State 2 (receiving docetaxel + ADT)	42.4 (18.0)	0.64 (0.27)
Base State 3 (completed docetaxel + on ADT; not progressed)	46.0 (18.5)	0.68 (0.26)
AEs (Base State 2 + specific AE)		
Fatigue	34.9 (16.8)	0.54 (0.34)
Nausea and vomiting	26.6 (15.4)	0.41 (0.36)
Reduced immunity	30.9 (15.8)	0.48 (0.33)
Fluid retention	36.7 (16.4)	0.58 (0.29)
Alopecia	33.4 (18.7)	0.58 (0.29)
Diarrhoea	25.6 (15.8)	0.40 (0.38)
Dead	3.9 (7.1)	

VAS values from 0 (“worst health”) to 100 (“best health”); TTO values from 0 (“equivalent to being dead”) to 1 (“perfect health”); a negative value would indicate a state worse than being dead

*ADT* androgen deprivation therapy, *AE* adverse event, *n* number, *SD* standard deviation, *TTO* time trade-off, *VAS* visual analogue scale

A small number of participants valued each health state as worse than dead. The AE state of diarrhoea grade 3/4 was rated as worse than dead by nine participants, which was more than any other AE state. Nausea and vomiting grade 3/4 was rated as worse than dead by eight participants, while fatigue grade 3 and reduced immunity grade 3/4 health states were rated this way by five participants (Description of health states available in the Supplementary Material).

### Regression model estimation of AE disutilities

The GEE model estimated health state utilities for each base state (Table 3). Base States 2 and 3 were significantly lower than Base State 1 (intercept = 0.71; CI 0.68, 0.75), with the utility decrement for Base State 2 estimated at − 0.07 (CI − 0.10, − 0.05) and for Base State 3 at − 0.04 (CI − 0.06, − 0.02). Utilities in Base States 2 and 3 were statistically significantly lower than that in Base State 1 (*p* = 0.0002 and *p* < 0.0001, respectively).

**Table 3:** Comparison of base health state utilities using a GEE model

Base states	Estimate	Standard error estimates	*p* value	Lower 95% CI	Upper 95% CI
Base State 1 (reference)	0.71	0.02		0.68	0.75
Base State 2	− 0.07	0.01	< 0.0001	− 0.10	− 0.05
Base State 3	− 0.04	0.01	0.0002	− 0.06	− 0.02

*CI* confidence interval, *GEE* generalized estimating equation

The AE model adjusted for sample covariates yielded the same estimate values for the AEs as the unadjusted model, and thus the model with the better fit (QIC_adjusted_ = 1410 vs. QIC_unadjusted_ = 1407) is presented (unadjusted) (Table 4). All AEs showed a significantly lower utility value when compared to Base State 2 (intercept = 0.37). Nausea and vomiting (Grade 3) had the largest impact (i.e. estimated disutility) on the transformed utility values (− 0.21), followed by diarrhoea (Grade 3) (− 0.18), reduced immunity (− 0.14), fatigue (− 0.09), fluid retention (Grade 3) (− 0.07), and alopecia (Grade 2) (− 0.04).

**Table 4:** Disutility estimates from the GEE model of AE health states from reference Base State 2 [estimated mean utility = 0.64 (SD 0.27)]

Adverse event	Estimate	Standard error estimates	*p* value	Lower 95% CI	Upper 95% CI
*Intercept*	*0.37*	*0.02*	< *0.0001*	*0.32*	*0.41*
Alopecia	− 0.04	0.01	0.0017	− 0.07	− 0.017
Diarrhoea	− 0.18	0.02	< 0.0001	− 0.22	− 0.14
Fatigue	− 0.09	0.02	< 0.0001	− 0.12	− 0.05
Fluid retention	− 0.07	0.01	< 0.0001	− 0.10	− 0.04
Nausea and vomiting	− 0.21	0.02	< 0.0001	− 0.25	− 0.17
Reduced immunity	− 0.14	0.02	< 0.0001	− 0.17	− 0.11

*AE* adverse event, *CI* confidence interval, *GEE* generalized estimating equation, *SD* standard deviation

## Discussion

This study elicited societal utility values for health states related to high-risk mHSPC and the disutility associated with treatment-related AEs using the TTO methodology with members of the UK general public. The results of the TTO scores indicated that Base State 1 was valued as the least impactful treatment state, while Base State 2, associated with docetaxel, was valued as the worst. Analyses also derived a range of disutility values for treatment-related AEs. The mean utility estimates showed that participants rated diarrhoea and nausea and vomiting as the most impactful on HRQL, while alopecia and fluid retention were considered the least burdensome.

The values from the present study fall within the range of published EQ-5D-5L utility values for metastatic prostate cancer patients which have been identified through systematic literature review (0.63–0.85) (37), which supports the validity of our approach. In addition, very recently, Chi et al. [28] reported an EQ-5D-5L-derived health utility value of 0.79 for patients with newly diagnosed high-risk mHSPC not yet treated in the LATITUDE study [28]. Furthermore, Lloyd et al. [15] collected HRQL data in men with mCRPC and the utility values elicited using the EQ-5D-5L ranged from 0.83 (asymptomatic/mildly symptomatic before docetaxel health state) to 0.63 (symptomatic before docetaxel health state). In the same study, utilities derived using the European Organisation for Research and Treatment of Cancer preference-based measure (EORTC-8D) were slightly higher and ranged from 0.70 to 0.86 for the same health states [15]. The patients who were currently receiving docetaxel had a mean EQ-5D-5L utility value of 0.69 and a mean EORTC-8D value of 0.75. The values are similar to those in the present study, despite Lloyd et al.’s focus on the mCRPC setting. Clinical trial data have also shown a reduction in HRQL for patients receiving docetaxel compared to ADT alone [13, 29, 30], as measured by the EORTC quality of life questionnaire (QLQ-C30) and functional assessment of cancer therapy-prostate (FACT-P) instruments, which supports the low utility values for Base State 2.

The GEE models estimated the difference in health utility between the base states and the disutility associated with each of the AEs. The GEE approach has advantages over the simple calculation of arithmetic means as a way of understanding the impact of AEs on HRQL. The analysis incorporates all data and so could be used to estimate values for combinations of states that were not captured in the study. This approach is also in line with good practice in the field where large trial datasets or observational data have attempted to tease apart the influence of different factors on HRQL [10].

The utility decrements for AEs from Base State 2 reflected a wide range of scores. Fluid retention and alopecia showed the smallest decrements as compared to other AEs. Fatigue had a higher utility decrement which is consistent with the burden associated with fatigue in this condition [4]. The disutilities for some of the AEs were quite large and reflect the severity of the states—for example, grade 3–4 diarrhoea or nausea/vomiting could lead to a hospital admission for fluid replacement. These AEs are particularly unpleasant and would significantly affect a patient’s HRQL. Grade 3–4 diarrhoea was associated with a decline in utility of -0.18; nausea/vomiting was − 0.21.

Measuring the impact of grade 3–4 adverse events in cancer patients is challenging because patients will often not feel well enough to complete surveys. This is one reason why teams may rely on vignette methods to estimate these effects.

There are some limitations related to the vignette approach, which are important to acknowledge. It is possible that the vignette methodology may not accurately reflect the extent to which patients learn to cope with and adjust to their disease. Vignettes can also lead people to overly focus on certain aspects of the description, could place undue weight on a specific descriptor, and may also pick up health effects not covered in a generic measure. For example, our states make reference to libido and sexual functioning, which are not mentioned in the EQ-5D-5L. Furthermore, the method of employing health state vignettes to elicit utilities has been criticised, partly because it is difficult to confirm the validity of the health states themselves [31]. The utilities derived from studies using this methodology rely heavily upon the accuracy of the descriptions developed and could be subject to bias.

In the current study, we aimed to maximise validity by using multiple sources of information such as information from the literature as well as from patient and clinician information interviews. The states also describe the main aspects of HRQL rather than solely focussing on prostate cancer-specific issues. Future research could be designed to measure HRQL using a standardised measure like EQ-5D-5L. However, it is worth considering that collecting HRQL data from patients experiencing severe grade 3–4 AEs, which may have an acute onset and could lead to hospitalisation, would be methodologically very challenging. The vignette methodology represents an alternative method for estimating the disutility of such difficult to assess health states.

Although it is generally recommended to use utilities from a single source for a given cost-effectiveness analysis, the methods used in this study have been used in others, so utilities for other health states are likely available. Future meta-analytic methods could be used to combine all reported published utilities and this paper could contribute to such an analysis. This study utilised the TTO method as preferred means of eliciting societal utility values; however, caution may be warranted when comparing utilities derived through TTO vs. other methods, such as standard gamble, since different valuation methods may elicit different utility values.

Comparison of our sample characteristics with available population data suggests it was broadly representative, although potentially over-represented younger people, females, those with higher education, non-white ethnic groups, and those in relatively good health. The available population demographic data were for England and Wales, whereas our study sample was drawn from England and Scotland. The additional GEE model we ran to test and adjust for participant’s age, gender, and EQ-5D index score as potential explanatory sample variables yielded the same estimate values for the AEs as the unadjusted model, which increases confidence in the limited influence of these sample characteristics.

## Conclusion

The study provides useful information on utility values for late-stage prostate cancer and the disutility of grade 3–4 AEs associated with two common treatments. These values provide insight into the perception of common side effects associated with the treatment of metastatic prostate cancer; they highlight the potential impact such events could have on patients’ HRQL and the importance of taking this into account in cost-effectiveness evaluation of treatments. These are the first set of AE disutilities to be generated for docetaxel in the mHSPC setting and may also be used to support economic evaluations of future treatments in mHSPC.

## Electronic supplementary material

Below is the link to the electronic supplementary material.

Supplementary material 1 (DOCX 44 KB)
