# Supplementary material for: Estimating utilities/disutilities for high-risk metastatic hormone-sensitive prostate cancer (mHSPC) and treatment-related adverse events
Source: Qual Life Res. 2019 Feb 14;28(5):1191–9. doi: 10.1007/s11136-019-02117-9 (PMC6470112; doi:10.1007/s11136-019-02117-9)
Supplement: Supplementary file 1 — Supplementary material 1 (DOCX 44 KB) [file 11136_2019_2117_MOESM1_ESM.docx]

**Supplementary material to**

Estimating Utilities / Disutilities For High Risk Metastatic Hormone Sensitive Prostate Cancer (mHSPC) And Treatment-Related Adverse Events.

Quality of Life Research

F. Hall*, HM. de Freitas, C. Kerr, T. Ito, B. Nafees, AJ. Lloyd, J. Penton, M. Hadi, S. Lanar, TP. Pham

* Janssen-Cilag, 50-100 Holmers Farm Way, High Wycombe, Bucks, HP12 4EG

email: fhall1@ITS.JNJ.com

**Health state descriptions**

| **Base Health State 1** |
| --- |
| *mHSPC: A newly diagnosed patient with high-risk* metastatic prostate cancer who is currently receiving ADT and is not yet castrate resistant.*  **High-risk disease is defined as having 2 of the following 3 risk factors: (1) Gleason score of ≥8; (2) presence of 3 or more lesions on bone scan; (3) presence of visceral metastasis.* |
| - You have recently been diagnosed with a serious illness. You are receiving treatment, either through daily tablets or a long-acting implant placed under the skin. - You sometimes experience hot flushes. You may experience tenderness and some swelling around your breast area. You have reduced sex drive and a reduced ability to have sexual relations. - You frequently have to pass urine, both during the day and at night. You often experience bone pain, particularly in your back and legs, which can be relieved by strong pain killers. - When you feel pain, it may lead to restricted movements, such as difficulty lying down/ getting up, walking around the house, and climbing stairs. The pain might make it difficult to focus on your work and it can cause poor sleep. When you feel pain, you may have some difficulty with washing and dressing yourself, doing jobs around the house, and shopping. - You are able to visit family and friends most of the time. You sometimes feel depressed about your condition. |

| **Base Health State 2** |
| --- |
| *Receiving docetaxel mHSPC: A patient with high-risk* metastatic prostate cancer who is currently receiving docetaxel plus ADT and is not yet castrate resistant.*  **High-risk disease is defined as having 2 of the following 3 risk factors: (1) Gleason score of ≥8; (2) presence of 3 or more lesions on bone scan; (3) presence of visceral metastasis.* |
| - You have a serious illness. You are receiving treatment, either through daily tablets or a long-acting implant placed under the skin. You also have to visit the hospital for a day once every three weeks to receive treatment via a drip. - You sometimes experience hot flushes. You may experience tenderness and some swelling around your breast area. You have reduced sex drive and a reduced ability to have sexual relations. - You frequently have to pass urine, both during the day and at night. You often experience bone pain, particularly in your back and legs, which can be relieved by strong pain killers. - When you feel pain, it may lead to restricted movements, such as difficulty lying down/ getting up, walking around the house, and climbing stairs. The pain might make it difficult to focus on your work and it can cause poor sleep. When you feel pain, you may have some difficulty with washing and dressing yourself, doing jobs around the house, and shopping. - You are only able to visit family and friends occasionally due to the risk of getting an infection. You sometimes feel depressed about your condition. |

| **Base Health State 3** |
| --- |
| *mHSPC post-docetaxel and pre-progression: A patient with high-risk* metastatic prostate cancer who has completed six cycles of docetaxel, who is currently on ADT and is not yet castrate resistant and has not yet progressed.*  **High-risk disease is defined as having 2 of the following 3 risk factors: (1) Gleason score of ≥8; (2) presence of 3 or more lesions on bone scan; (3) presence of visceral metastasis.* |
| - You have a serious illness. You are receiving treatment, either through daily tablets or a long-acting implant placed under the skin. You have recently completed additional treatment which required you to visit the hospital for a day once every three weeks to receive treatment via a drip. - You sometimes experience hot flushes. You may experience tenderness and some swelling around your breast area. You have reduced sex drive and a reduced ability to have sexual relations. - You frequently have to pass urine, both during the day and at night. You often experience bone pain, particularly in your back and legs, which can be relieved by strong pain killers. - When you feel pain, it may lead to restricted movements, such as difficulty lying down/ getting up, walking around the house, and climbing stairs. The pain might make it difficult to focus on your work and it can cause poor sleep. When you feel pain, you may have some difficulty with washing and dressing yourself, doing jobs around the house, and shopping. - You are able to visit family and friends most of the time. You often feel depressed about your condition. |

*Note that for all six AEs, the AE description is integrated within the wording of Base State 2.*

| **AE state 1** |
| --- |
| *Fatigue*  *Grade 3; Fatigue not relieved by rest; limiting self-care ADL* |
| - You have a serious illness. You are receiving treatment, either through daily tablets or a long-acting implant placed under the skin. You also have to visit the hospital for a day once every three weeks to receive treatment via a drip. - You sometimes experience hot flushes. You may experience tenderness and some swelling around your breast area. You have reduced sex drive and a reduced ability to have sexual relations. - You frequently have to pass urine, both during the day and at night. You often experience bone pain, particularly in your back and legs, which can be relieved by strong pain killers. - You experience tiredness and feel lethargic most of the time. - When you feel pain or tiredness, it may lead to restricted movements, such as difficulty lying down/ getting up, walking around the house, and climbing stairs. The pain or tiredness might make it difficult to focus on your work and it can cause poor sleep. When you feel pain or tiredness, you have difficulty with washing and dressing yourself, doing jobs around the house, and shopping. - You are rarely able to visit family and friends because you lack the energy to leave the house and due to the risk of getting an infection. You sometimes feel depressed about your condition. |

| **AE state 2** |
| --- |
| *Nausea and vomiting*  *Grade 3-4*  *Nausea: Inadequate oral caloric or fluid intake; IV fluids, tube feedings, or TPN indicated ≥24 h*  *Vomiting: ≥6 episodes in 24h; IV fluids, or TPN indicated ≥24 h* |
| - You have a serious illness. You are receiving treatment, either through daily tablets or a long-acting implant placed under the skin. You also have to visit the hospital for a day once every three weeks to receive treatment via a drip. - You sometimes experience hot flushes. You may experience tenderness and some swelling around your breast area. You have reduced sex drive and a reduced ability to have sexual relations. - You frequently have to pass urine, both during the day and at night. You often experience bone pain, particularly in your back and legs, which can be relieved by strong pain killers. - You experience nausea and vomiting occasionally which lasts 2–3 days. During those days, you feel very nauseous and vomit intermittently. You receive treatment for the vomiting which could mean going into hospital for a few days. - When you feel pain or experience nausea and vomiting, it may lead to restricted movements, such as difficulty lying down/ getting up, walking around the house, and climbing stairs. The pain or the nausea and vomiting might make it difficult to focus on your work and it can cause poor sleep. When you feel pain or experience nausea and vomiting, you have difficulty with washing and dressing yourself, doing jobs around the house, and shopping. - You are rarely able to visit family and friends because of the nausea and vomiting and due to the risk of getting an infection. You often feel depressed about your condition. |

| **AE State 3** |
| --- |
| *Reduced immunity & higher susceptibility to infections*  *Grade 3-4; Neutrophils <1000/mm3 to 500/mm3 or Neutrophils <500/mm3* |
| - You have a serious illness. You are receiving treatment, either through daily tablets or a long-acting implant placed under the skin. You also have to visit the hospital for a day once every three weeks to receive treatment via a drip. - You sometimes experience hot flushes. You may experience tenderness and some swelling around your breast area. You have reduced sex drive and a reduced ability to have sexual relations. - You frequently have to pass urine, both during the day and at night. You often experience bone pain, particularly in your back and legs, which can be relieved by strong pain killers. - A blood test has indicated that your immune system is low and therefore you are much more likely to get an infection. - When you feel pain, it may lead to restricted movements, such as difficulty lying down/ getting up, walking around the house, and climbing stairs. The pain might make it difficult to focus on your work and it can cause poor sleep. When you feel pain, you may have some difficulty with washing and dressing yourself, doing jobs around the house, and shopping. - Because of the risk of infection, you are rarely able to visit family and friends, you may avoid public transport, go out less, and restrict social activities to avoid being around people. You sometimes feel depressed about your condition. |

| **AE State 4** |
| --- |
| *Fluid Retention*  *Grade 3; >30% inter-limb discrepancy in volume; gross deviation from normal anatomic contour, limiting self-care ADL* |
| - You have a serious illness. You are receiving treatment, either through daily tablets or a long-acting implant placed under the skin. You also have to visit the hospital for a day once every three weeks to receive treatment via a drip. - You sometimes experience hot flushes. You may experience tenderness and some swelling around your breast area. You have reduced sex drive and a reduced ability to have sexual relations. - You frequently have to pass urine, both during the day and at night. You often experience bone pain, particularly in your back and legs, which can be relieved by strong pain killers. - You experience swelling in your hands, legs, ankles and feet which causes some discomfort. - When you feel pain or discomfort, it may lead to restricted movements, such as difficulty lying down/ getting up, walking around the house, and climbing stairs. The pain or discomfort might make it difficult to focus on your work and it can cause poor sleep. When you feel pain or discomfort, you may have difficulty with washing and dressing yourself, doing jobs around the house, and shopping. - You are only able to visit family and friends occasionally due to the risk of getting an infection. You sometimes feel depressed about your condition. |

| **AE State 5** |
| --- |
| *Alopecia*  *Grade 2: Hair loss of ≥50 percent normal for that individual that is readily apparent to others; a wig or hair piece is necessary if the patient desires to completely camouflage the hair loss; associated with psychosocial impact* |
| - You have a serious illness. You are receiving treatment, either through daily tablets or a long-acting implant placed under the skin. You also have to visit the hospital for a day once every three weeks to receive treatment via a drip. - You sometimes experience hot flushes. You may experience tenderness and some swelling around your breast area. You have reduced sex drive and a reduced ability to have sexual relations. - You frequently have to pass urine, both during the day and at night. You often experience bone pain, particularly in your back and legs, which can be relieved by strong pain killers. - You have lost your hair. - When you feel pain, it may lead to restricted movements, such as difficulty lying down/ getting up, walking around the house, and climbing stairs. The pain might make it difficult to focus on your work and it can cause poor sleep. When you feel pain, you may have some difficulty with washing and dressing yourself, doing jobs around the house, and shopping. - You are only able to visit family and friends occasionally due to the risk of getting an infection. You sometimes feel depressed about your condition. |

| **AE State 6** |
| --- |
| *Diarrhoea*  *Grade 3-4*  *Grade 3: Increase of seven or more stools per day over baseline; incontinence; hospitalisation indicated; severe increase in ostomy output compared with baseline; limiting self-care activities of daily living*  *Grade 4: Life-threatening consequences; urgent intervention indicated* |
| - You have a serious illness. You are receiving treatment, either through daily tablets or a long-acting implant placed under the skin. You also have to visit the hospital for a day once every three weeks to receive treatment via a drip. - You sometimes experience hot flushes. You may experience tenderness and some swelling around your breast area. You have reduced sex drive and a reduced ability to have sexual relations. - You frequently have to pass urine, both during the day and at night. You often experience bone pain, particularly in your back and legs, which can be relieved by strong pain killers. - You have diarrhoea several times a day which means you frequently need to go to the toilet urgently, and experience severe abdominal cramps. You may need to go to hospital to be re-hydrated. - When you feel pain or have diarrhoea, it may lead to restricted movements, such as difficulty lying down/ getting up, walking around the house, and climbing stairs. The pain or diarrhoea might make it difficult to focus on your work and it can cause poor sleep. When you feel pain or have diarrhoea, you have difficulty with washing and dressing yourself, doing jobs around the house, and shopping. - You are only able to visit family and friends occasionally because of the diarrhoea and due to the risk of getting an infection. You sometimes feel depressed about your condition. |

**Literature review search strategies**

**OVID MEDLINE DATABASE**

| **Terms** | **Keywords** | **Commands** |
| --- | --- | --- |
| 1. HRQOL | (health-related quality of life ***OR*** health related quality of life ***OR*** HRQL ***OR*** HRQOL ***OR*** quality of life ***OR*** QOL ***OR*** burden).mp ***OR*** (Quality of Life) [MeSH] | AND |
| 2. Qualitative research | (qualitative ***OR*** phenomenolog* ***OR*** grounded theory ***OR*** focus group* ***OR*** interview* ***OR*** endpoint model* ***OR*** conceptual ***OR*** thematic ***OR*** patient experience).mp ***OR*** (Qualitative research ***OR*** Interview ***OR*** focus groups) [MeSH] | AND |
| 3. Prostate cancer | metastatic prostate cancer.mp ***OR*** (prostatic neoplasms) [MeSH] | AND |
| 4. Androgen deprivation therapy | androgen deprivation therapy.mp ***OR*** (Androgen Antagonists) [MesH] | AND |
| 5. Chemotherapy | Chemotherapy.mp | OR |

mp = title, abstract, original title, name of substance word, subject heading word, keyword heading word, protocol supplementary concept word, rare disease supplementary concept word, unique identifier

**OVID EMBASE DATABASE**

| **Terms** | **Keywords** | **Commands** |
| --- | --- | --- |
| HRQOL | (health-related quality of life ***OR*** health related quality of life ***OR*** HRQL ***OR*** HRQOL ***OR*** quality of life ***OR*** QOL ***OR*** burden).mp ***OR*** (Quality of Life) [MeSH] | AND |
| Qualitative research | (qualitative ***OR*** phenomenolog* ***OR*** grounded theory ***OR*** focus group* ***OR*** interview* ***OR*** endpoint model* ***OR*** conceptual ***OR*** thematic ***OR*** patient experience).mp ***OR*** (Qualitative research ***OR*** Qualitative analysis ***OR*** Semi structured interview ***OR*** Interview) [MeSH] | AND |
| Prostate cancer | metastatic prostate cancer.mp ***OR*** (Metastasis ***AND*** Prostate Cancer) [MeSH] | AND |
| Androgen deprivation therapy | androgen deprivation therapy.mp ***OR*** (Antiandrogen ***OR*** Androgen Deprivation Therapy) [MesH] | AND |
| Chemotherapy | Chemotherapy.mp ***OR*** (Cancer chemotherapy ***OR*** Chemotherapy) [MeSH] | OR |

mp = title, abstract, heading word, drug trade name, original title, device manufacturer, drug manufacturer, device trade name, keyword, floating subheading

**OVID PSYCHINFO DATABASE**

| **Terms** | **Keywords** | **Commands** |
| --- | --- | --- |
| HRQOL | (health-related quality of life ***OR*** health related quality of life ***OR*** HRQL ***OR*** HRQOL ***OR*** quality of life ***OR*** QOL ***OR*** burden).mp ***OR*** (Quality of Life) [MeSH] | AND |
| Qualitative research | (qualitative OR phenomenolog* ***OR*** grounded theory ***OR*** focus group* ***OR*** interview* ***OR*** endpoint model* ***OR*** conceptual ***OR*** thematic ***OR*** patient experience).mp ***OR*** (Qualitative research ***OR*** interviews ***OR*** group discussion) [MeSH] | AND |
| Prostate cancer | metastatic prostate cancer.mp ***OR*** (prostate ***AND*** neoplasms) [MeSH] | AND |
| Androgen deprivation therapy | androgen deprivation therapy.mp | AND |
| Chemotherapy | Chemotherapy.mp ***OR*** (chemotherapy) [MeSH] | OR |

mp = title, abstract, heading word, table of contents, key concepts, original title, tests & measures
